# Self-Detachment and Subsurface Densification of Dealloyed Nanoporous Thin Films

**DOI:** 10.1021/acs.nanolett.2c02666

**Published:** 2022-08-11

**Authors:** Gideon Henkelmann, Diana Waldow, Maowen Liu, Lukas Lührs, Yong Li, Jörg Weissmüller

**Affiliations:** † Institute of Materials Physics and Technology, Hamburg University of Technology, 21073 Hamburg, Germany; ‡ Institute of Materials Mechanics, Helmholtz-Zentrum Hereon, 21502 Geesthacht, Germany

**Keywords:** nanoporous metals, dealloying, coarsening, surface diffusion, Plateau-Rayleigh instability, nanowires

## Abstract

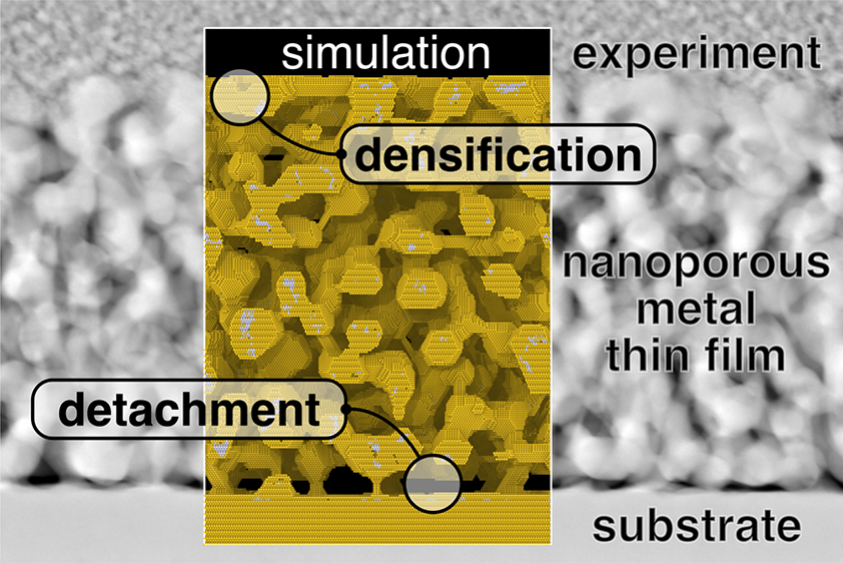

Experiment shows thin films of dealloyed nanoporous gold (NPG) spontaneously detaching from massive gold base layers. NPG can also densify near its external surface. This is naturally reproduced by kinetic Monte Carlo (KMC) simulation of dealloying and coarsening and so appears generic for nanoscale network materials evolving by surface diffusion. Near the porous layer’s external surface and near its interface with the base layer, gradients in the depth-profile of a laterally averaged mean surface curvature provide driving forces for diffusion and cause divergences of the net fluxes of matter, leading to accretion/densification or to erosion/disconnection. As a toy model, the morphology evolution of substrate-supported nanopillars by surface diffusion illustrates and confirms our considerations. Contrary to cylindrical nanowires, the ligaments in nanoporous materials exhibit pre-existing gradients in the mean curvature. The Plateau-Rayleigh long-wavelength stability criterion is then not applicable and the disconnection accelerated.

Nanoporous gold (NPG) is formed by the self-organization processes that act during dealloying,^1,2^ a corrosion process that selectively removes one element from a homogeneous solid solution as the master alloy. At the corrosion front, primary dealloying^3^ generates the nanoscale network structure as a result of the competition between the advance of the corrosion by dissolution and of a trend for passivation when surface diffusion forms an atomic monolayer of the more noble element.^4−7^ Behind the corrosion front, the much slower process of secondary dealloying is brought about by curvature-driven and surface-diffusion mediated coarsening. This process exposes further less-noble element, previously buried underneath the surface, to dissolution.^3,8−10^ The pinch-off of ligaments by Plateau-Rayleigh-like instabilities is the topology-changing event during coarsening.^5,9^ While those processes have been established, essential features of the resulting microstructure, for instance, the size of the struts (“ligaments”) of the network for given corrosion conditions, cannot yet be predicted.^10^ The origin of the denser skin layer near the external surface of nanoporous metal samples^11,12^ also awaits clarification. Here, based on an investigation of nanoporous thin films, we expose how the processes that act during dealloying lead to densification near free surfaces and to decohesion near the interface with a massive substrate.

NPG exhibits interesting mechanical and functional properties.^13^ The mainstream of the related studies explores free-standing bulk samples, millimeters in size in all three dimensions. Yet, substrate-supported thin films of NPG are under study and may provide opportunities for integrating the material into functional elements or devices,^14−19^ specifically as cantilever actuators^20,21^ and sensors,^22^ as substrates for surface-enhanced Raman scattering,^23^ for biomedical applications^24,25^ or studies of optical properties,^26−28^ energy-storage,^29^ electro-catalysis^30^ and photoenhanced catalysis.^31^

The adhesion to the substrate is a concern for thin films of any material.^32^ Master samples for thin films of NPG have the substrate covered by few nm of adhesion layer, e.g., Cr, Ti or Nb, followed by few tens of nm of pure Au and finally by the AgAu master alloy layer proper.^14,15,33^ The Au layer is to ensure that the porous material terminates, at its bottom, in a dense blanket that acts as a baseplate, anchors the film to the substrate, and prevents attack of the adhesion layer.

Our study shows that the just-mentioned strategy will, contrary to the intention and to the intuition, in fact systematically impair adhesion. It promotes the formation, during secondary dealloying or upon aging, of a low-density decohesion layer between the porous film and the blanket. This provides an example of a more general phenomenon, where gradients in an in-plane averaged mean curvature entail a divergence of the out-of-plane mass transport during coarsening of nanoscale pore structures. That divergence, in turn, entails local erosion or accretion of matter, thereby disconnecting or densifying the local ligament network. Specifically, we show that this mechanism also underlies the formation of the dense skin layer near the external surface of NPG.

Nanoporous thin-film samples were made by dealloying DC magnetron sputtered Ag–Cu thin films, see Methods in the Supporting Information (SI). Inspection in the scanning electron microscope regularly revealed a gap with only remarkably loose connectivity between the massive gold base layer and the nanoporous film proper. Figure 1a exemplifies this finding by means of a TEM image of one of our samples. The dark regions between the gold base layer and the NPG film clearly show the partial decohesion. Sensitized by this observation, we found quite similar defects in micrographs of earlier published reports^19,25,33,34^ on NPG films, even though none of those studies addresses the feature. Figure 1b,c exemplifies such earlier observations, here from refs (34) and (33), respectively.

**Figure 1:**
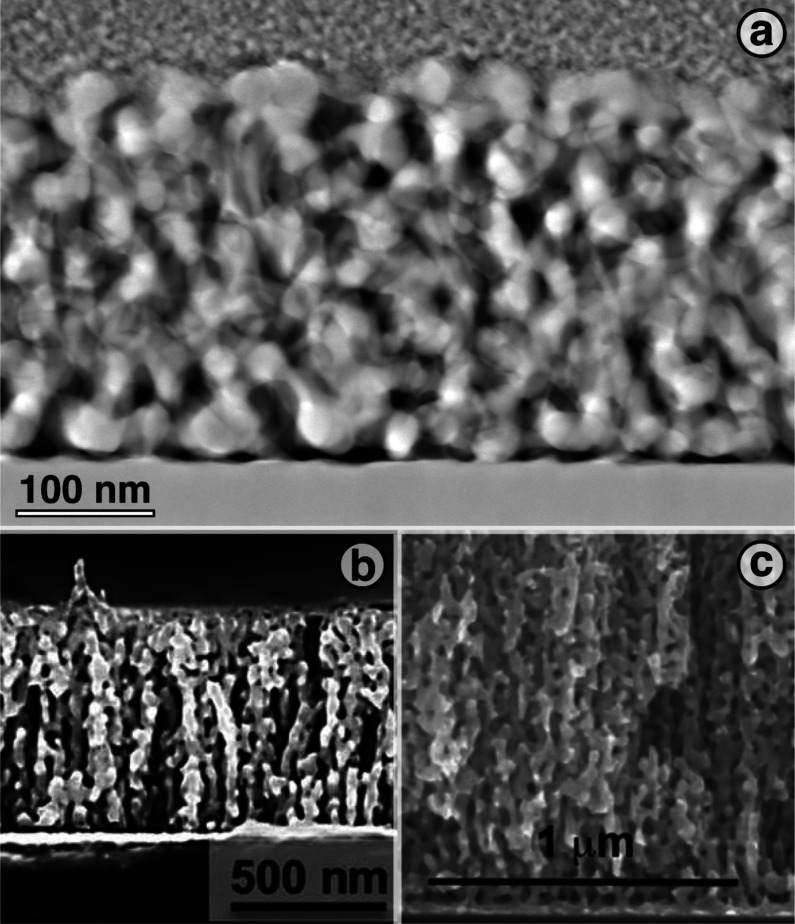
Experimental observations of low connectivity near the base of nanoporous gold (NPG) thin films. (a) High-angle annular dark-field transmission electron microscopy image of a focused-ion-beam cut lamella from an as-prepared film; this work. Pt coating is visible as a granular contrast on top, Au substrate as a solid block at bottom, NPG as porous structure in center. Dark gap between substrate and NPG indicates low connectivity. (b) Scanning electron microscopy (SEM) image of dealloyed Au_36_Ag_64_ 660 nm thin film on top of a 80 nm Au adhesion layer; detail from a figure by Kurtulus et al.^34^ (c) SEM image of dealloyed Au_32_Ag_68_ 1300 nm thin film on top of a 30 nm Au adhesion layer; detail from a figure by Okman and Kysar.^33^ Note layers of low connectivity at bottom in (b,c). Panel (b) reprinted in part with permission from ref (34). Copyright 2014, Royal Society of Chemistry. Used with permission. Panel (c) reprinted in part with permission from ref (33). Copyright 2011, Elsevier. Used with permission.

KMC simulations of thin film dealloying, see Methods in the SI, were run in order to clarify the underlying processes. Simulations with different electrode potential parameters, ϕ, and different master alloy layer composition, *x*_Au_, and thickness all provided consistent results. Here, we display and discuss results obtained with *x*_Au_ = 0.25 and ϕ = 1.1 V. The ϕ-value provided dissolution current density and time for completion of primary dealloying roughly consistent with the experiment.

The evolution of the solid volume fraction, φ, with time, *t*, during the simulation is shown in Figure 2. Videos S1, S2, S3 show further details, and Figure S1 shows the evolution of the *x*_Au_ depth profile near the interfaces magnified. Dealloying starts at the top, to the right in Figure 2, and appears as a decrease in φ, already apparent in the graph for 1.1 s, that works its way inward (to the left and to lower values of position, *z*), away from the external surface.

**Figure 2:**
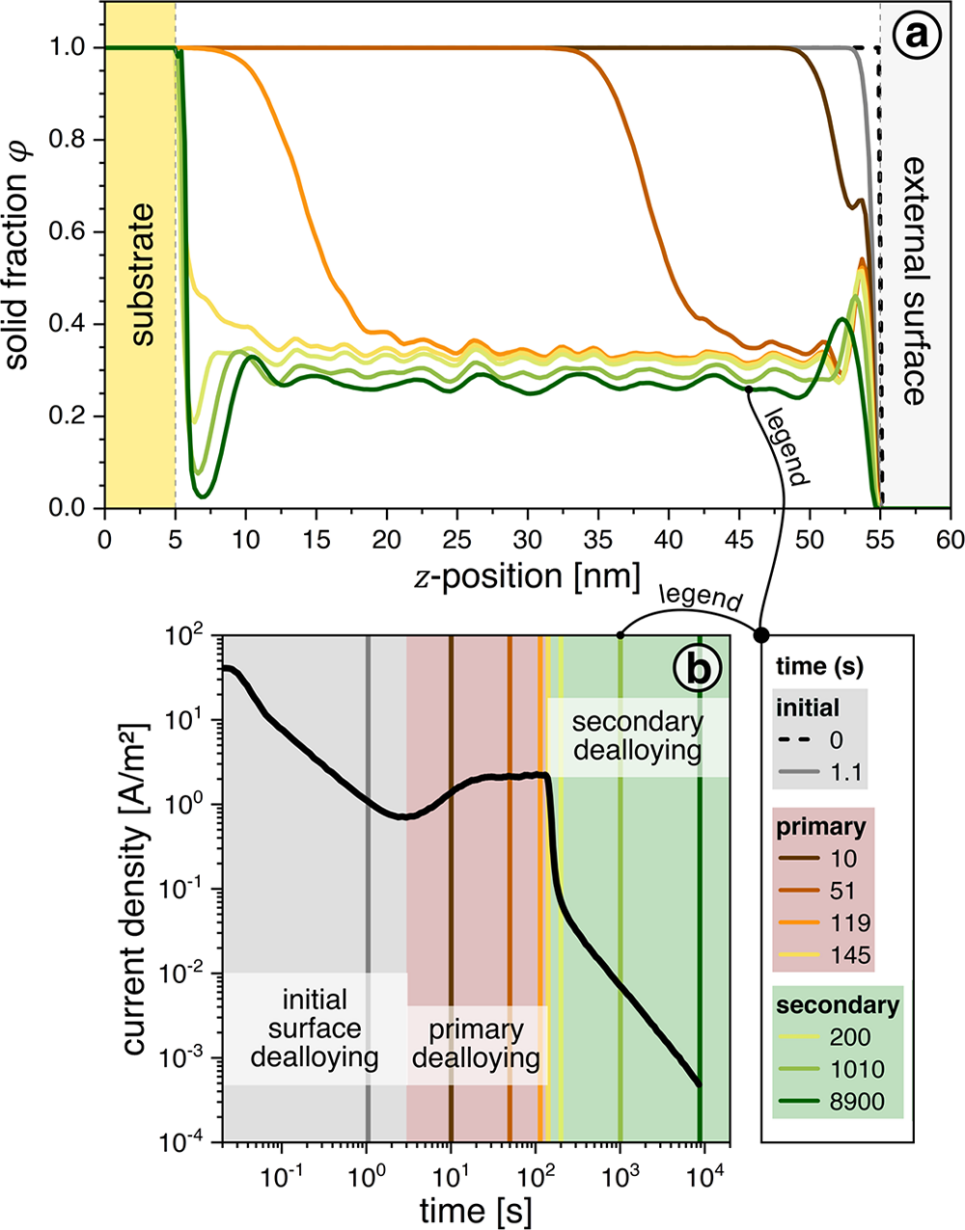
Kinetic Monte Carlo simulation of dealloying. (a) Graphs of solid fraction, φ, versus position in *z*-direction (normal to the plane of the film). Snapshots at different moments in apparent time, as indicated by legend. Note how the dealloying front, marked by the drop in φ, moves inward (from right to left) with time and how a region of low φ, indication of incipient decohesion, then develops near the substrate. (b) Current density versus apparent time, averaged over 100 samples from separate simulation runs. Note the transition from primary to secondary dealloying, as marked by steep drop in current density, after 1.5 × 10^2^ s.

The graphs for 3 s < *t* < 150 s follow the progression of the corrosion front into the depth. The density enhancement in the top layer becomes more pronounced over time, and the mean surface position (where φ drops to zero) moves slightly inward. Both observations indicate a net inward flux of Au near the surface.

The corrosion front for bulk dealloying takes about 120 s for reaching the substrate. At and beyond 150 s, a plateau of low φ, marking the porous solid, extends almost throughout the entire domain of the original alloy film. At this instant, the dissolution current (frequency of Ag removal events) was observed to drop off and diffusion-related events to become dominant. This marks the completion of primary dealloying.^10^

During secondary dealloying, the extra density in the top layer smears out over a thicker region. Simultaneously, the density profile near the contact with the base layer develops a pronounced dip. This marks the onset of film detachment. After ∼10^4^ s, the lateral average φ at the center of the dip approaches zero; in other words, detachment is almost complete. Note that the base layer, which was completely exposed after 150 s, has by now again been covered with a thin layer of dense material; this points to a net flux of matter out of the porous regions toward the base layer.

Figure 3 compares the experimental microstructure of an as-dealloyed film (left-hand-side column) to that of the final stage of the KMC simulation of dealloyed thin films (right-hand-side column). The experiment probed a focused-ion-beam-cut lamella with EDS imaging. The top and bottom rows of Figure 3 show microstructure renderings and in-plane averaged volumetric density profiles of the constituents, respectively. The density profiles for the experiment are specified as EDS counts, and the profiles for the simulation are specified as fractional occupancy (by any species, Ag or Au) of lattice sites, averaged laterally over the simulation box.

**Figure 3:**
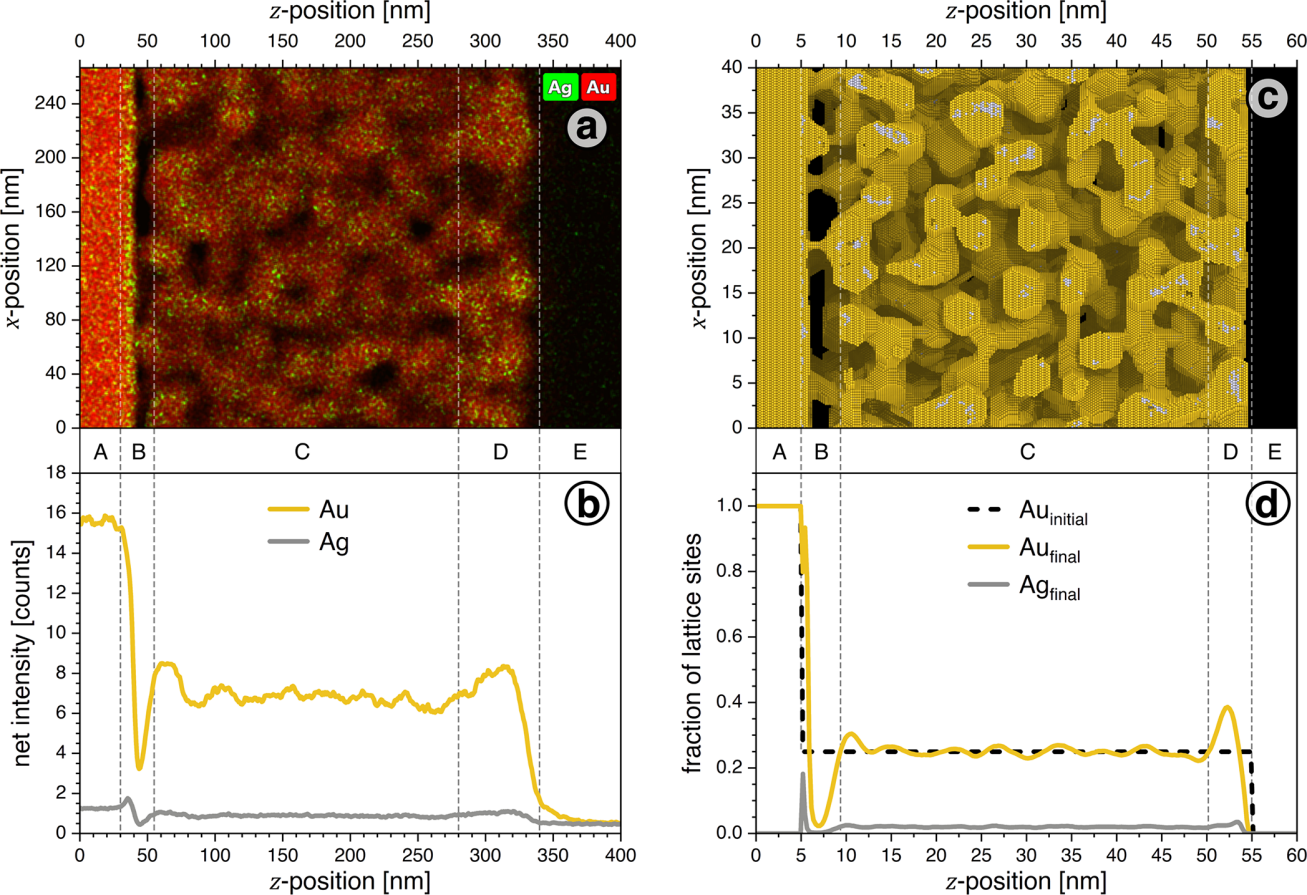
Comparing experimental and simulation results for the averaged in-plane densities of Au and Ag. (a) Elemental maps of Au (red) and Ag (green) from experimental energy-dispersive xray spectroscopy (EDS). Sample of Figure 1a, substrate on the left and open surface on the right. (b) Net intensity, averaged along perpendicular slices of the EDS map. (c) Rendering of the simulated microstructure after 9 × 10^3^ s of apparent time. (d) Fraction of occupied lattice sites by element, averaged over 100 samples from separate simulation runs. Note that net intensity in the experimental EDS map and fractional site occupancy in the simulation results both indicate volumetric density of the respective element. Labels A–E denote distinct regions as discussed in the main text.

According to the elemental density profiles, five layers (A–E from left/bottom to right/top) can be distinguished in Figure 3:

- The pure Au base layer, (A).
- The region, (B), of low density and low connectivity bordering on the base layer. Note that experiment and simulation agree on this feature. Between substrate and NPG, the experiment shows a 10 nm gap with 50% reduced net density. The simulation suggests that the solid fraction in the gap is as low as 0.02, as compared to 0.25 in the bulk of the porous film. A substantial loss of connections can be observed in both renderings. In both data sets, note also the small but significant peaks in the Ag density right at the base layer. That feature suggests that several crystal lattice planes at the very bottom of the original Au_25_Ag_75_ master alloy are only partially dealloyed and get buried under multiple layers of Au. Since bulk diffusion is negligible under the conditions of our experiment and simulation, such buried features are conserved until they are exposed to corrosion during the later stages of the microstructure evolution.^8^
- The homogeneous nanoporous thin film, (C). The ligament sizes in experiment and simulation are 19 ± 4 and 4 ± 2 nm, respectively. Buried Ag-rich regions (see above) can be observed in the simulation rendering.
- The densified region, (D), near the surface of the porous film. Experiment and simulation here agree on a surface layer of increased density. In the simulation, the surface has moved inward by 1 ± 0.5 nm compared to the initial Au_25_Ag_75_ alloy. This points to a net flux of matter from the external surface into the porous regions, well consistent with the inward-displacement of the external surface and the enhanced solid fraction right underneath.
- The initial outer environment, (E), unaffected by dealloying.

Experiment and simulation agree on (i) the deficit in density and connectivity near the base and (ii) the enhanced density near the surface. Apparently, the simulation catches the essential physics behind those phenomena.

In an idealized scenario, we now explore the underlying mechanism qualitatively and within a picture that is coarse-grained at a scale larger than the ligament size. We focus on secondary dealloying or on postdealloying aging/annealing, where the microstructure evolution is dominated by coarsening.

The microstructure evolution of network materials such as NPG is mediated by surface diffusion that is driven by gradients in the chemical potential, μ. The Gibbs-Thomson equation relates the local value of μ to magnitude and sign of the local mean curvature, κ, by
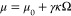
1Here, γ, Ω, and μ_0_ denote the surface tension, the atomic volume and the chemical potential of the homogeneous bulk phase, respectively. We use κ in its definition as the sum of the inverse principal radii of the solid’s surface. Matter tends to be redistributed away from more convex surface segments and toward less convex or more concave ones.^35−37^

Motivated by the experiment, we consider a laterally extended planar sheet of the network material with its upper surface in contact to vacuum and its lower one attached to a dense base layer of the same phase as the network struts (here, massive gold). We consider a 1D transport problem along the *z*-direction, normal to the sheet. An effective chemical potential, μ̅(*z*), is obtained by eq 1 with κ replaced by its lateral (in the plane of the sheet) average, κ̅(*z*). Within this coarse-grained scenario, the lateral average diffusion flux, *j̅* (atoms per macroscopic film footprint area per time), is driven by gradients in μ̅ according to
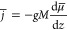
2where *M* is a mobility coefficient and *g* is a geometry factor that depends on ligament size, phase fraction, φ = ρ̅/ρ_0_ (where ρ̅ is the laterally averaged density of matter and ρ_0_ its value in the massive material), and strut size. Furthermore, we consider a continuity equation that links the time-dependent mass balance in any volume element to gradients in the flux
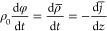
3Equations 2 and 3 link the evolution of the solid fraction with time to the second derivative of the mean curvature
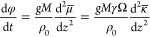
4For our argument we find it sufficient to discuss eq 4 with attention to the initial diffusion fluxes, at the onset of coarsening of the network material.

As an illustrative scenario that may serve as a toy model, we explore an array of massive, cylindrical gold pillars of radius *r*_0_ that terminate in hemispherical tips at the top and that are attached, at the bottom, to a contiguous base layer of massive gold. The schematic in Figure 4a illustrates the geometry of a selected pillar. Figure 4b shows how κ varies, initially, along one of the pillars. The curvature is at maximum in the tip, which is biaxially curved and strongly convex, κ ∼ +2/*r*_0_. Intermediate positive values, κ = +1/*r*_0_, prevail along the uniaxially curved central regions of the shafts. Finally, the region where the pillars merge into the base layer exhibits oppositely signed principal radii and, hence, small positive or even, as in the example, negative κ.

**Figure 4:**
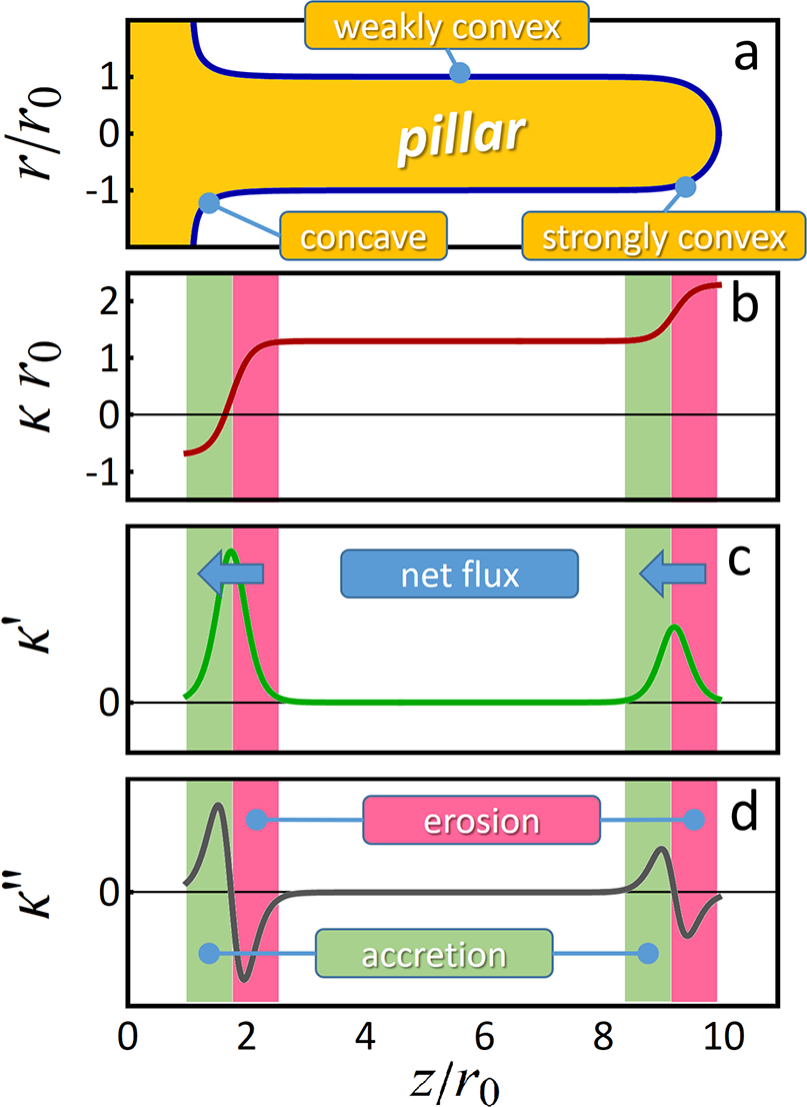
Schematic representation of model for boundary effects on the density evolution in porous microstructures. (a) Cross-section of the model geometry, showing pillar (yellow, shaded) of radius *r*_0_ with convex tip at its outer surface (to the right) and concave regions at its contact with the base layer. Local envelope radius *r* versus axial coordinate *z*. (b) Variation of mean curvature, κ, along the *z*-axis. (c) Gradient, κ′, of κ along the *z*-axis. According to eq 2, regions of the surface with positive κ′ prompt flux of matter, by surface diffusion, to the left as indicated. (d) Second derivative, κ″, of κ along the *z*-axis. According to eq 4, regions of the surface with positive κ″ experience accretion of matter, while negative κ″ leads to erosion. Note specifically regions with erosion near the bottom of the pillar and at its very top. The arguments that suggest the erosion near the bottom are equally applicable to the disconnection observed at the base of nanoporous thin films.

A qualitatively similar depth profile of κ is expected in a network material. Specifically, the termination of the ligaments at the outer surface requires strong convexity. The complementary situation prevails at the interface with the dense base layer—here, pore channels terminate and this requires strongly concave surfaces. The network structure in the interior of NPG exhibits convex, concave, and saddle-shaped regions. The average of its mean curvature, while positive valued, is substantially smaller than 1/*R* (with *R* the characteristic radius of the ligaments).^38−41^ These arguments imply that the curvature gradients in the toy model—strong convexity at the top, week convexity in the center, concave surfaces at the bottom—reflect qualitatively the situation in substrate-supported films of NPG.

By virtue of eq 2, gradients of the mean curvature drive the diffusion fluxes illustrated by arrows in Figure 4c, and eq 4 implies accretion or erosion as suggested by the colored regions in Figure 4d. The inward transport at the outer boundary (top of the pillars) lets the position of that boundary move inward while simultaneously thickening the pillars somewhat further down. In the network material, that effect corresponds to a densification in a layer just underneath that surface. By contrast, there is a region of flux divergence near the bottom, where fluxes directed toward the convex contact region increasingly deplete the adjacent part of the shaft from material. This results in necking, the analog of the detachment of the network material near its base layer.

For verification, we have explored the evolution of nanopillars by surface diffusion in a KMC simulation. Figure 5 shows snapshots at different moments in time. For additional details, see the Video SV4. Figure 5 is for a (111)-oriented pillar; similar results were found for other orientations. A trend for faceting is seen, as is—most importantly—an ever increasing deviation from the original, cylindrical size. The first pronounced variation is precisely the one predicted by the above analysis–necking near the base layer, and thickening near the tip. At a somewhat later stage, the central shaft of the pillar develops the series of additional necks that is characteristic of the Plateau-Rayleigh instability.^42−44^ The initial necking and thickening near top and bottom of the pillar develop much earlier than the onset of the Plateau-Rayleigh instability, since they are driven by pre-existing curvature gradients. The gradients that drive the fluxes of the Plateau-Rayleigh instability become relevant only after a substantial incubation time.

**Figure 5:**
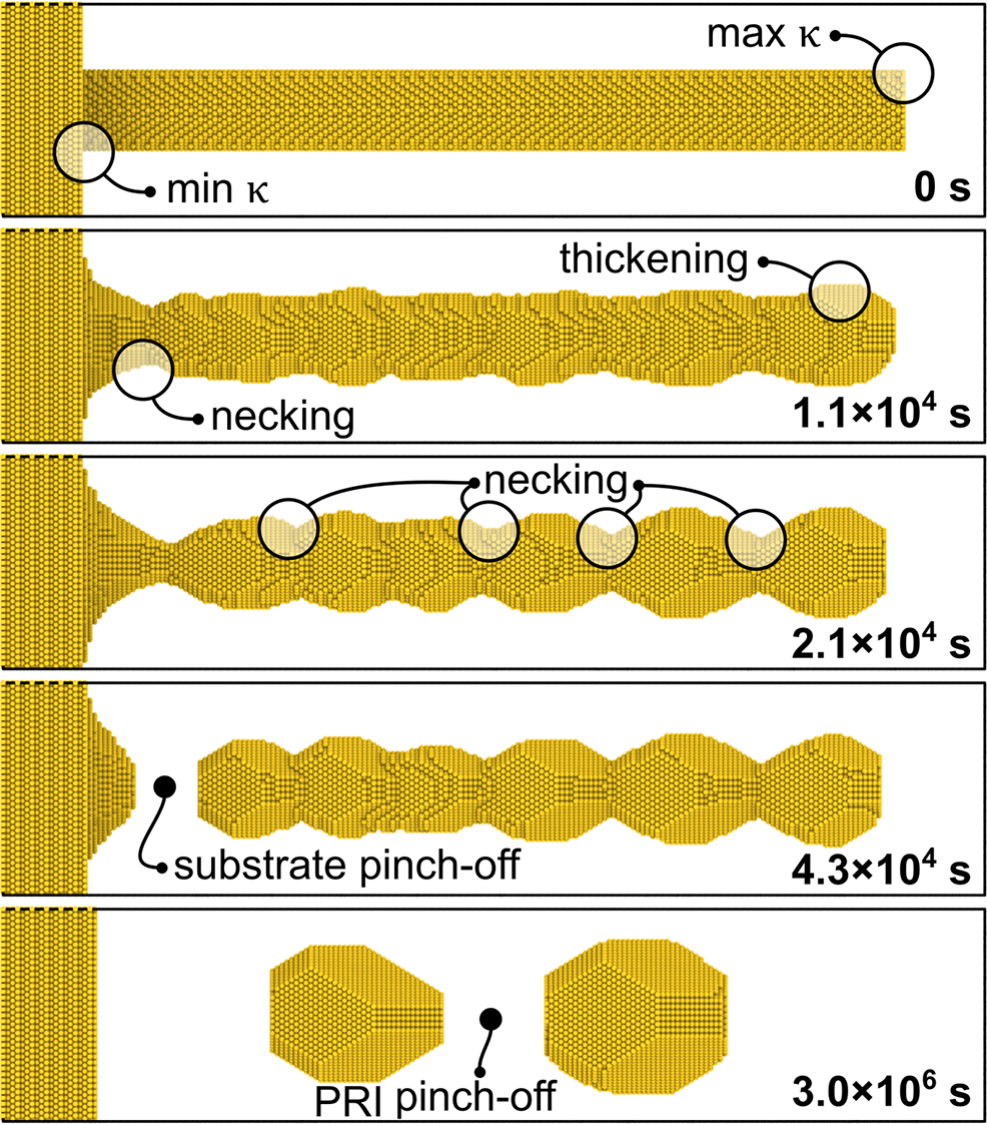
KMC simulation of microstructure evolution by surface diffusion for a substrate-supported cylindrical Au nanopillar. Initial radius 2 nm and height 40 nm. Pillar axis is 111-oriented. Snapshots at five consecutive stages of the evolution are shown; bold labels indicate apparent time. Circles mark distinctive features, as explained by labels, including regions of minimal and maximum initial curvature, κ. Bottom two panels show first pinch-off at the substrate and after very much longer time pinch-off by the Plateau-Rayleigh instability (PRI).

Clearly, the implications of the toy model, as outlined above, are in excellent qualitative agreement with (1) the observations from our experiments, with (2) the findings from our atomistic simulation, and with (3) our qualitative analysis of the driving forces and diffusion fluxes and their verification in the toy model. Thus, we conclude that the toy model’s discussion correctly captures the essential physics behind our observations.

Disconnections at the interface between the porous layer and the base material, as well as the pinch-off of ligaments of NPG during coarsening, have parallels to the Plateau-Rayleigh instability. Yet, our observations highlight important differences. The Plateau-Rayleigh instability requires that a small perturbation of the shape of an initially cylindrical body reduces the net surface energy. In an isotropic fluid, this is only possible when the wavelength of the perturbation exceeds the circumference of the cylinder.^45^ In a solid with anisotropic surface tension, the instability may even be completely suppressed at any wavelength.^46^ The processes of necking and disconnection in nanoporous metals differ because of the pre-existing gradients in the mean curvature. These gradients provide driving forces for erosion or accretion, which may prompt disconnections at short wavelength and in spite of anisotropy.

In support of the above notion, the long-wavelength restriction is relaxed in fluids once an initial modulation with a finite amplitude has formed.^47^ Furthermore, the growth kinetics and topology evolution in nanoporous gold are not qualitatively affected when, at elevated temperature, the surface roughens and the surface tension becomes more isotropic.^9^ Studies of Plateau-Rayleigh instabilities in Au nanowires reveal the fast breakup at wire junctions, where there are pre-existing curvature gradients.^44^ This is observed at low temperatures, where the free-standing cylindrical regions of the nanowires remain stable. That observation is in keeping with models for the detachment of secondary dendrite arms from a dendrite trunk in alloy solidification.^48^ Results of a phase-field simulation (with isotropic surface energy) of that process are closely consistent with our KMC simulation,^49^ as is the observation that the spheroidization of dendrite arms is accelerated near the convex tips.^50^

To summarize, our study explores the trend for densification or disconnection near planar interfaces in nanoporous solids. These processes are observed in experiments with thin films of dealloyed nanoporous gold, and they are naturally reproduced by kinetic Monte Carlo simulations of dealloying. Gradients in a laterally averaged mean curvature of the local pore surfaces emerge as the origin of densification or disconnection. Those gradients are a natural consequence of the microstructural geometry of the porous layer: the termination of ligaments at the macroscopic external surface requires strongly convex surfaces whereas the termination of pore channels at the interface with a massive substrate requires strongly concave surfaces. By the fact that disconnections in the porous material are driven by pre-existing curvature gradients, they do not connect one-to-one to the stability criteria and kinetics of the Plateau-Rayleigh instability.

One implication of the above consideration is that a standard strategy for enhancing the adhesion of dealloyed thin films of nanoporous gold to the substrate may not be ideally appropriate: somewhat counterintuitively, planar massive base layers of gold inherently favor decohesion. One is led to ask about mitigation strategies. Work in progress in our team suggests that smoothing out the composition profile transition between the gold base layer and the Ag–Au master alloy film may reduce the trend for decohesion. As the planarity of the base layer is connected to the origin of the observation, one may also speculate that a roughened base layer might bring further benefits. Such strategies, if confirmed, could enhance the applicability of dealloyed porous metal films in the various applications addressed in this work’s introduction.

## Supporting Information Available

Full description on methods as well as the details of the elemental density evolution, including Figure S1 and citing further references (PDF)
Video showing perspective view of microstructure evolution during the dealloying of Figure 2; each video shows a sequence of snapshots recorded at times *t*_*j*_ in the simulation time frame, with *t*_*j*_ = *τ t*_*j*–1_ and τ = 1.05, and in the viewer’s time frame the video acceleration increases exponentially with time (MP4)
Video showing side view of microstructure evolution during the dealloying of Figure 2; each video shows a sequence of snapshots recorded at times *t*_*j*_ in the simulation time frame, with *t*_*j*_ = *τ t*_*j*–1_ and τ = 1.05, and in the viewer’s time frame the video acceleration increases exponentially with time (MP4)
Video showing top view of microstructure evolution during the dealloying of Figure 2; each video shows a sequence of snapshots recorded at times *t*_*j*_ in the simulation time frame, with *t*_*j*_ = *τ t*_*j*–1_ and τ = 1.05, and in the viewer’s time frame the video acceleration increases exponentially with time (MP4)
Video showing microstructure evolution during the nanopillar breakup of Figure 5; each video shows a sequence of snapshots recorded at times *t*_*j*_ in the simulation time frame, with *t*_*j*_ = *τ t*_*j*–1_ with τ = 1.1 for SV4, and in the viewer’s time frame the video acceleration increases exponentially with time (MP4)
