# Supplementary material for: Self-Detachment and Subsurface Densification of Dealloyed Nanoporous Thin Films
Source: Nano Lett. 2022 Aug 11;22(16):6787–93. doi: 10.1021/acs.nanolett.2c02666 (PMC9413411; doi:10.1021/acs.nanolett.2c02666)
Supplement: Supplementary file 1 — nl2c02666_si_001.pdf [file nl2c02666_si_001.pdf]

Supplementary Information for "Self-Detachment and Sub-Surface Densification of Dealloyed Nanoporous Thin Films"

By Gideon Henkelmann, Diana Waldow, Maowen Liu, Lukas Lührs, Yong Li and Jörg Weissmüller

Hamburg University of Technology, Helmholtz-Zentrum Hereon

Methods

Experiment

Precursors were prepared in a multi-source DC magnetron sputtering setup with a base pressure of 10^{-7} mbar. Silicon wafers, (100)-oriented, 100 µm thick and with a 100 nm thick thermally grown oxide layer (Si-Mat Silicon Materials) were cleaned with an Ar ion beam in the load lock and then coated at room temperature. An adhesive Ti layer was deposited first, followed by the pure Au base layer. Subsequently, $Au_{25}Ag_{75}$ was deposited from an alloy target. For the thicknesses of the layers, transmission electron microscopy (TEM) analysis shows 5 ± 1 nm of Ti, 55 ± 1 nm of Au and 302 ± 9 nm of NPG.

Dealloying was performed similar to Ref. [1] in 1 M $HClO_4$ at 0.75 V vs. Ag/AgCl pseudo-reference (1.27 V vs. the standard hydrogen electrode). A steep drop of the dealloying current after ~ 10 min marked the completion of primary dealloying. After the current decreased to below 20 µA (at ~ 27 min), the electrical potential was increased to 0.85 V for further Ag removal; that potential was held for another ~ 45 min. In fresh electrolyte, 3 potential cycles were applied, ranging from -0.4 to 1.1 V at a scan rate of $5\,\mathrm{mV\,s^{-1}}$, ending at 0.4 V, which corresponds to an oxygen-free surface. The samples were rinsed in ultrapure water followed by EtOH before drying in vacuum.

For transmission electron microscopy (TEM) sample preparation, the porous films were first infiltrated (at 100 °C for 1 h) with phthalic anhydride-based mounting wax (Gatan) in order to support the network structure. After depositing a Pt layer on top of a film, a lamella was prepared by focused Ga-ion beam milling (FEI Helios G3 UC) and transferred to a Cu grid.

The lamella, with the final thickness of around 100 nm in the area of interest, was characterized by a FEI Talos F200X TEM equipped with an energy-dispersive X-ray spectroscopy (EDS) detector system operating at an accelerating voltage of 200 kV. High-angle annular dark-field (HAADF) images and EDS maps were taken using a probe current of 1 nA.

Simulation

An on-lattice kinetic Monte Carlo (KMC) simulation approach was used for modeling dealloying and coarsening. The crystal lattice was generated by filling a cuboid simulation volume element with a lattice of face-centered cubic unit cells, oriented for a (111) top surface. Periodic boundary conditions applied in the plane parallel to that surface. The bottom 5 nm (in z-direction, aligned with the surface normal) were filled with Au atoms to represent the base layer, while the top 5 nm were left empty modeling the open surface. The remaining volume – $\sim 40 \times 40\,\mathrm{nm}^2$ in cross-section and ~ 50 nm thick comprising $\sim 4.8 \times 10^6$ atoms – was randomly filled with Ag and Au so as to generate a random $Au_{25}Ag_{75}$ solid solution.

Consistent with earlier work [2–6], the kinetics was restricted to two type of events: dissolution (Ag atoms disappear) and diffusion (Ag or Au atoms jump to neighboring vacancies). Only atoms with 9 or fewer neighbors were active for either, dissolution or diffusion. Diffusion target sites were restricted to vacancies with at least 3 neighbors (including the diffusing atom). Rates of diffusion, k_{dif}, and dissolution, k_{dis}, were provided by the bond breaking model of [2],

$$k_{\mathrm{dif}} = \nu_{\mathrm{dif}} \exp\left(-\frac{nE_{\mathrm{b}}}{k_{\mathrm{B}}T}\right) \tag{S1}$$

$$k_{\mathrm{dis}} = \nu_{\mathrm{dis}} \exp\left(-\frac{nE_{\mathrm{b}} - e\phi}{k_{\mathrm{B}}T}\right) \tag{S2}$$

with diffusion attempt frequency ν_{dif}, dissolution attempt frequency ν_{dis}, next neighbor count n, bond energy E_{b}, Boltzmann constant k_{B}, Temperature T, elementary charge e and electrode potential parameter ϕ. The parameter values were adopted from earlier work [2], specifically $\nu_{\mathrm{dif}} = 10^{13}$ Hz, $\nu_{\mathrm{dis}} = 10^4$ Hz, $E_{\mathrm{b}} = 0.15$ eV. The simulation of microstructure evolution in nanowires considered a pure Au starting material and diffusion only, omitting the dissolution steps. All simulations used $T = 300$ K.

For each step, the physical time is increased by

$$\Delta t = -\frac{\ln c}{\sum_i k_i} \tag{S3}$$

with a random number c in the range $(0, 1]$ and the rate sum of all possible events $\sum_i k_i$ [7].

The implementation of the algorithm was built upon previous work of Li [5, 8]. Images of the resulting lattice were rendered using the Tachyon ray tracer integrated inside the Ovito scientific visualization software [9].

Intermediate configurations of the simulation were saved to disk at regular intervals and that data was evaluated for extracting composition profiles and the dissolution current transient. The finite number of sampling

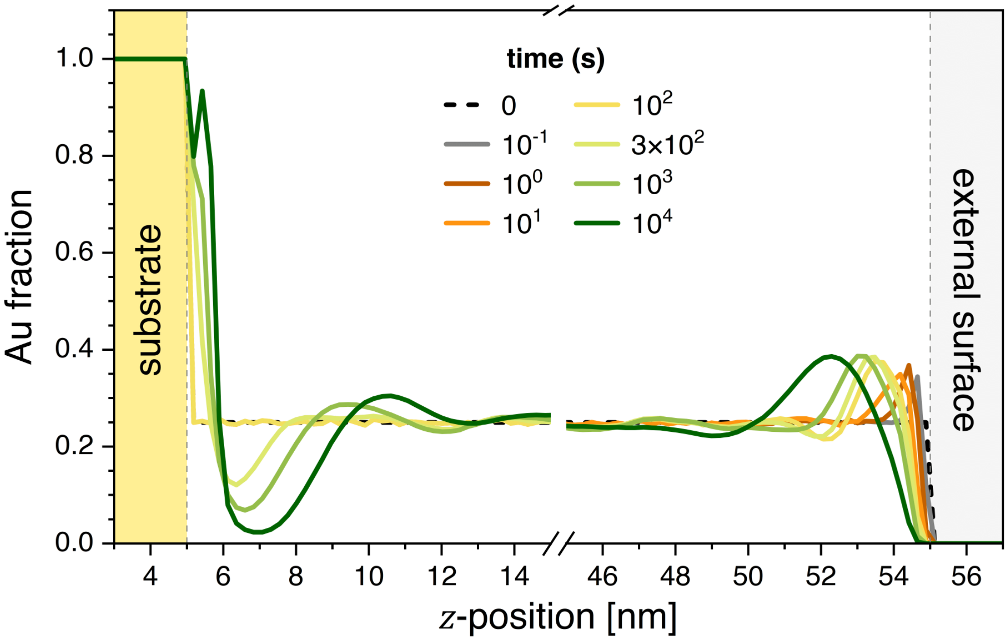

Figure S1: Time evolution of Au fraction – defined as the lateral average of the fraction of simulation-box lattice sites occupied by Au – for the dealloying simulation of Fig 2. Note axis break on the abscissa, permitting a focus on the regions near the interfaces.The top layer, marked by a peak in Au fraction, retracts and thickens over time. After the completion of primary dealloying at around 10^2 s, the substrate grows at the expense of the nanoporous thin film, resulting in continuously increasing detachment. As in Fig 2, the data represents an average over 100 simulation runs for the identical scenario.

points and the discreteness of the porous microstructure both create scatter in the resulting graphics. For a representative display, the entire KMC simulation for each scenario was repeated, typically 100 times, and the graphs of dissolution current and the density profiles computed as averages over those repeated runs.

Details of elemental density evolution

In the main article, Fig 2 shows the simulated time evolution of the solid fraction. Figure S1 highlights localized features near the interfaces. Note the axis break, which allows a higher depth-resolution in the regions of interest. Note also that the graphs display the Au-fraction rather than the net (Ag and Au) atom density. After 10^4 s, the substrate has thickened by $\sim$ 1 nm and the top layer has moved inwards by $\sim$ 1 nm.

Bibliography

References

[1] Lührs, L.; Soyarslan, C.; Markmann, J.; Bargmann, S.; Weissmüller, J. Elastic and plastic Poisson's ratios of nanoporous gold. *Scripta Materialia* **2016**, *110*, 65–69.

[2] Erlebacher, J. An Atomistic Description of Dealloying. *Journal of The Electrochemical Society* **2004**, *151*, C614.

[3] Erlebacher, J. Mechanism of Coarsening and Bubble Formation in High-Genus Nanoporous Metals. *Physical Review Letters* **2011**, *106*, 225504.

[4] Krekeler, T.; Straßer, A. V.; Graf, M.; Wang, K.; Hartig, C.; Ritter, M.; Weissmüller, J. Silver-rich clusters in nanoporous gold. *Materials Research Letters* **2017**, *5*, 314–321.

[5] Li, Y.; Ngo-Dinh, B.-N.; Markmann, J.; Weissmüller, J. Evolution of length scales and of chemical heterogeneity during primary and secondary dealloying. *Acta Materialia* **2022**, *222*, 117424.

[6] Artymowicz, D.; Erlebacher, J.; Newman, R. Relationship between the parting limit for de-alloying and a particular geometric high-density site percolation threshold. *Philosophical Magazine* **2009**, *89*, 1663–1693.

[7] Andersen, M.; Panosetti, C.; Reuter, K. A Practical Guide to Surface Kinetic Monte Carlo Simulations. *Frontiers in Chemistry* **2019**, *7*, 202.

[8] Li, Y.; Ngô, B.-N. D.; Markmann, J.; Weissmüller, J. Topology evolution during coarsening of nanoscale metal network structures. *Physical Review Materials* **2019**, *3*, 076001.

[9] Stukowski, A. Visualization and analysis of atomistic simulation data with OVITO–the Open Visualization Tool. *Modelling and Simulation in Materials Science and Engineering* **2009**, *18*, 015012.
